# ﻿*Altimella* gen. nov., a new genus of Cicurinidae (Arachnida, Araneae) from Xizang, China, with description of two new species

**DOI:** 10.3897/zookeys.1210.127910

**Published:** 2024-08-22

**Authors:** Lu-Yu Wang, Yan-Nan Mu, Yong-Qiang Xu, Feng Zhang, Zhi-Sheng Zhang

**Affiliations:** 1 Key Laboratory of Eco-environments in Three Gorges Reservoir Region (Ministry of Education), School of Life Sciences, Southwest University, Chongqing 400715, China Southwest University Chongqing China; 2 Institute of Plateau Biology of Xizang Autonomous Region, Lhasa 850001, Xizang Autonomous Region, China Institute of Plateau Biology of Xizang Autonomous Region Lhasa China; 3 Medog Biodiversity Observation and Research Station of Xizang Autonomous Region, Medog, China Medog Biodiversity Observation and Research Station of Xizang Autonomous Region Medog China; 4 The Key Laboratory of Zoological Systematics and Application, Institute of Life Science and Green Development, College of Life Sciences, Hebei University, Baoding, Hebei 071002, China Hebei University Baoding China

**Keywords:** description, distribution, morphology, taxonomy, Tibet

## Abstract

*Altimella* Wang & Zhang, **gen. nov.**, a new genus belonging to Cicurinidae, is established, and two new species are described, *Altimellanedong* Wang & Zhang, **sp. nov.** (♂♀, type species) and *A.ngamring* Wang & Zhang, **sp. nov.** (♂♀), from Xizang, China. Detailed descriptions of somatic features and genital characteristics, photos of the habitus, photos and drawings of the copulatory organs, and a distribution map are provided.

## ﻿Introduction

The spider family Cicurinidae F.O. Pickard-Cambridge, 1893 was restored and three genera (*Cicurina* Menge, 1871, *Brommella* Tullgren, 1948, and *Chorizomma* Simon, 1872) were confirmed by Gorneau et al. (2023). Currently, the type genus, *Cicurina*, comprises 144 species, most of which are distributed in North America and Asia, but with a few recorded from Europe. The genus *Brommella* includes 22 species, of which 17 have been reported from China, one from South Korea and Japan, three from the USA, and two from Europe. The third genus, *Chorizomma*, found in Spain and France, is monotypic and was once considered a subgenus of *Cicurina* (WSC 2024).

Gorneau et al. (2023) proposed synapomorphies for Cicurinidae: the absence of a cribellum, replaced by a reduced colulus with several setae; three tarsal claws, and legs without scopulae or claw tufts; the male palp with a variable retroventral tibial apophysis (RvTA); and the RTA enlarged (in some cases as long as the cymbium length), usually with an RTA-conductor. However, some Chinese *Brommella* species like *B.baiseensis* Li, 2017 and *B.casseabri* Li, 2017 possess obvious cribella (Li and Wang 2017: figs 6D, 8D).

We describe a new genus to accommodate two new species of Cicurinidae from Xizang, China, which also possess a cribellum like *Brommella* species, but they differ from *Brommella* by having a longer retrolateral tibial apophysis.

## ﻿Materials and methods

All specimens are preserved in 75% ethanol and were examined, illustrated, photographed, and measured using a Leica M205A stereomicroscope equipped with a drawing tube, a Leica DFC450 Camera, and LAS v. 4.6 software. Male palps and epigynes were examined and illustrated after they were dissected. Epigynes were cleared by immersing them in pancreatin for about 1 h (Álvarez-Padilla and Hormiga 2007). Eye sizes were measured as the maximum dorsal diameter. Leg measurements are shown as: total length (femur, patella and tibia, metatarsus, tarsus). All measurements are in millimeters. Specimens examined here are deposited in the Collection of Spiders, School of Life Sciences, Southwest University, Chongqing, China (**SWUC**).

Abbreviations used in the text: **ALE** = anterior lateral eye; **AME** = anterior median eye; **PLE** = posterior lateral eye; **PME** = posterior median eye; **RTA** = retrolateral tibial apophysis.

## ﻿Taxonomy

**Family Cicurinidae F.O. Pickard-Cambridge, 1893**

### ﻿ Altimella

Taxon classificationAnimaliaAraneaeCicurinidae

Wang & Zhang gen. nov.

8A299F90-F06F-5AF7-B360-D0B9B35F8E53

https://zoobank.org/22CB1EAD-C4FC-4860-8C5F-F98F21ABC763

#### Type species.

*Altimellanedong* Wang & Zhang, sp. nov.

#### Diagnosis.

*Altimella* Wang & Zhang, gen. nov. species resemble those of *Brommella* (Li and Wang 2017: 126, figs 1–61) in having an undivided cribellum, a large, simple retrolateral tibial apophysis with a single fold (Figs 2C, 3E, 4C, 5E), a tibial apophysis (spur) arising from the base of the tibia retro-ventrally, a long and filiform embolus, and long and complexly winding copulatory ducts, but they differ from *Brommella* by the long retrolateral tibial apophysis, as long as the cymbium (Figs 2B, C, 3D, E, 4B, C, 5D, E; vs shorter than the cymbium), the simple distal part of the conductor (Figs 2B, 3D, 4B, 5D; vs complex, spiral-shaped, or with three branches), and the obvious spermathecae (Figs 2E, 3G, 4E, 5G; vs inconspicuous).

**Figure 1.**
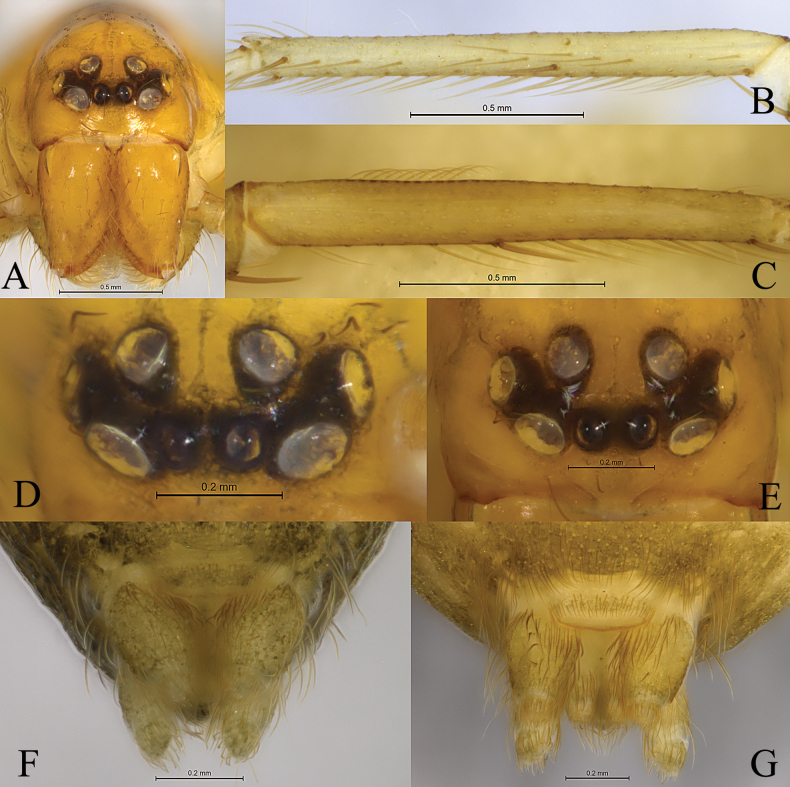
*Altimellanedong* Wang & Zhang, sp. nov. **B, D, F** holotype male **A, C, E, G** paratype female **A** male eyes and chelicerae, frontal view **B, C** metatarsus of Leg I, lateral view **D, E** eyes, front view **F, G** cribellum ventral view.

**Figure 2.**
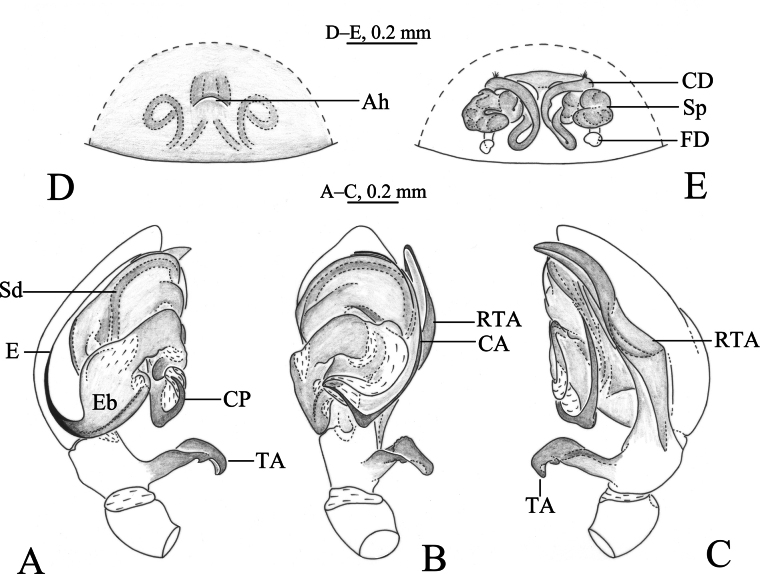
*Altimellanedong* Wang & Zhang, sp. nov. **A–C** holotype male **D, E** paratype female **A** left male palp, ventral view **B** same, retrolateral view **C** same, dorsal view **D** epigyne, ventral view **E** epigyne, dorsal view. Abbreviations: CA = anterior arm of conductor; CD = copulatory duct; CO = copulatory opening; CP = posterior arm of conductor; E = embolus; Eb = embolic base; FD = fertilization duct; RTA = retrolateral tibial apophysis; Sd = sperm duct; Sp = spermathecal; TA = tibial apophyses.

**Figure 3.**
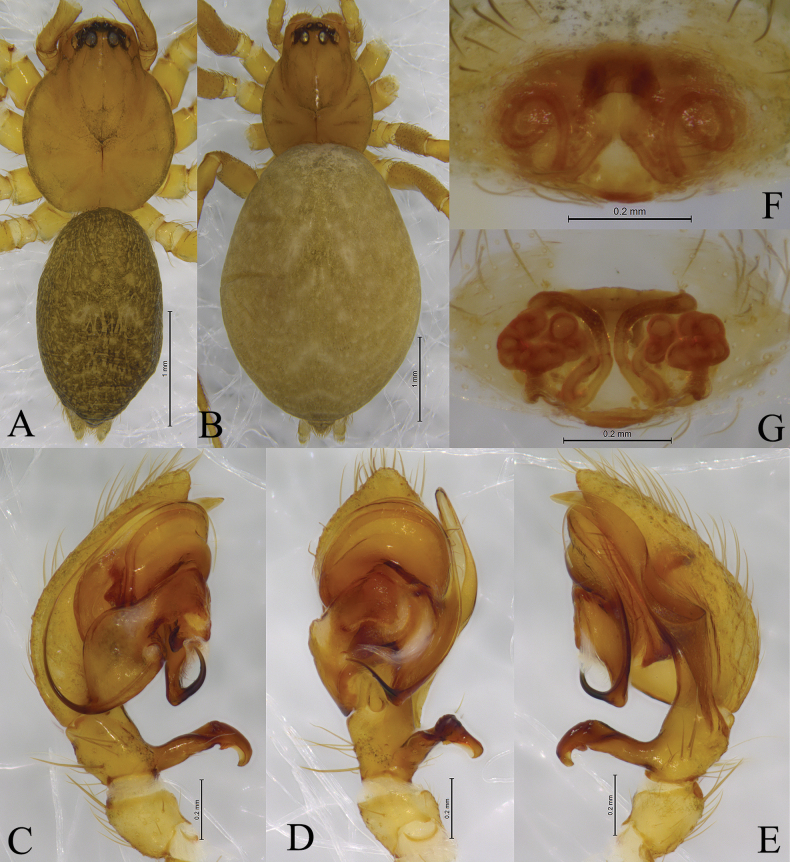
*Altimellanedong* Wang & Zhang, sp. nov. **A, C–E** holotype male **B, F, G** paratype female **A** male habitus, dorsal view **B** female habitus, dorsal view **C** left male palp, ventral view **D** same, retrolateral view **E** same, dorsal view **F** epigyne, ventral view **G** same, dorsal view.

**Figure 4.**
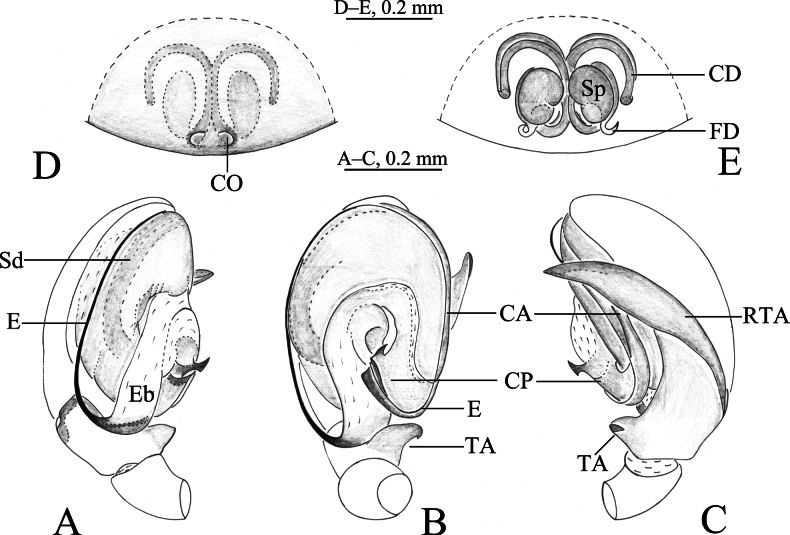
*Altimellangamring* Wang & Zhang, sp. nov. **A–C** holotype male **D, E** paratype female **A** left male palp, ventral view **B** same, retrolateral view **C** same, dorsal view **D** epigyne, ventral view **E** epigyne, dorsal view. Abbreviations: CA = anterior arm of conductor; CD = copulatory duct; CO = copulatory opening; CP = posterior arm of conductor; E = embolus; Eb = embolic base; FD = fertilization duct; RTA = retrolateral tibial apophysis; Sd = sperm duct; Sp = spermathecal; TA = tibial apophyses.

**Figure 5.**
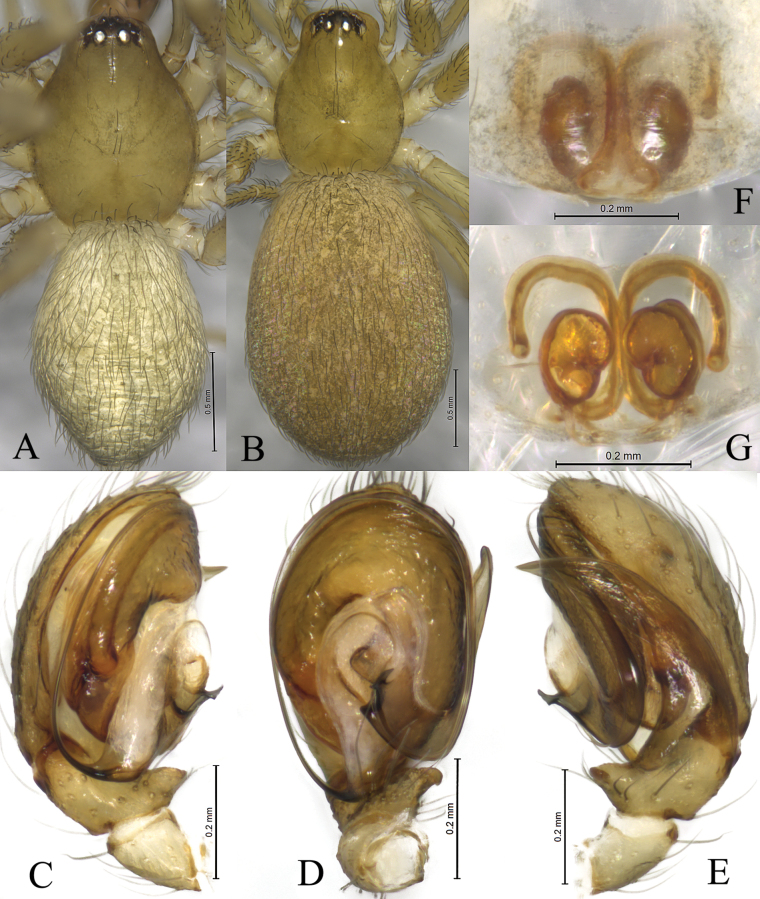
*Altimellangamring* Wang & Zhang, sp. nov. **A, C–E** holotype male **B, F, G** paratype female **A** male habitus, dorsal view **B** female habitus, dorsal view **C** left male palp, ventral view **D** same, retrolateral view **E** same, dorsal view **F** epigyne, ventral view **G** same, dorsal view.

#### Etymology.

The generic name is composed of the prefix “*alti*-” (high) and the suffix “-*mella*” (from *Brommella*), referring to the high-altitude type locality and similarity to *Brommella*. The gender is feminine.

#### Description.

Small size (male (*n* = 2): 2.30–3.55, female (*n* = 11): 2.70–5.19). Carapace yellowish brown to brown. Eight eyes (Fig. 1A, D, E). Cervical groove and radial furrows distinct. Chelicerae yellowish brown, with 3 promarginal and 3–4 retromarginal teeth. Labium and endites yellowish brown. Sternum yellowish brown and scutellate with sparse black setae. Legs yellowish brown. Calamistrum absent in male and weak (10 setae) in female (Fig. 1B, C). Opisthosoma oval, dorsum and venter yellowish brown, with small (about 0.2 mm) and undivided cribellum (Fig. 1F, G). Leg formula: 1423 or 4123.

***Male palp*** (Figs 2A–C, 3C–E, 4A–C, 5C–E): tibia with two apophyses, ventral (TA) and retrolateral (RTA): ventral lobe originates from base of tibia, longer than tibia, slightly bent near tip; retrolateral apophysis as long cymbium, with fold along entire apophysis. Bulb longer than wide. Anterior part of tegulum rounded, posterior part hidden by conductor (C), sperm duct (Sd) narrow, running along margin of tegulum; conductor with two arms, posterior claw-like (CP) and anterior spine-like (CA); embolus with large base (Eb), long and filiform, originating at 6–7 o’clock position, its anterior part resting in a long groove of anterior part of conductor.

***Epigyne*** (Figs 2D, E, 3F, G, 4D, E, 5F, G): epigynal plate wider than long or as long as wide. Copulatory openings wide, located anteriorly or posteriorly. Copulatory ducts long and complex, winding. Spermathecae convoluted or kidney-shaped. Fertilization ducts hook-like.

#### Composition.

Comprises two new species, *A.nedong* Wang & Zhang, sp. nov. and *A.ngamring* Wang & Zhang, sp. nov.

#### Biology.

Found in high altitude areas of the Qinghai-Tibet Plateau (Xizang). They construct sheet webs under stones.

#### Distribution.

**Figure 6.**
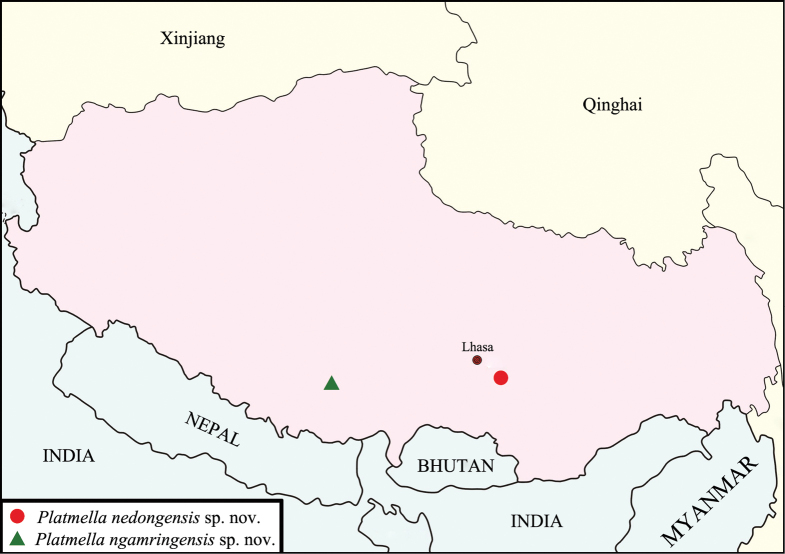
Distribution of *Altimella* in China.

China (Xizang) (Fig. 6).

#### Remarks.

This is the fourth genus of Cicurinidae and has a cribellum similar to species of *Brommella*. The epigynes of all four cicurinid genera are very similar, while male palps have more differences. Additionally, *Cicurina* and *Chorizomma* do not have cribella, while *Brommella* and *Altimella* gen. nov. do.

### ﻿ Altimella nedong

Taxon classificationAnimaliaAraneaeCicurinidae

Wang & Zhang sp. nov.

F07F97F9-413C-50E9-84FC-FBB79C5C6B74

https://zoobank.org/205BC42B-3D4A-43A3-9EEF-AA43AB0384F7

Figs 1 , 2 , 3 , 6 乃东高朗蛛

#### Type material.

***Holotype*** ♂ (SWUC-T-CI-08-01), China, Xizang, Shannan City, Nedong District, Zedang Town, 29°13'56′′N, 91°46'36′′E, elev. 3616 m, 25.08.2002, M.S. Zhu leg. (SWUC). ***Paratypes***: 6♀ (SWUC-T-CI-08-02 to 07) with same data as for holotype (SWUC) • 2♀ (SWUC-T-CI-08-08 to 09), Zedang Town, 21.08.2002, F.X. Liu leg.

#### Etymology.

The specific name is derived from the type locality; a noun in apposition.

#### Diagnosis.

The new species differs from those of *A.ngamring* Wang & Zhang, sp. nov. (Figs 4, 5) by the ventral tibial apophysis as long as tibia (Figs 2A–C, 3C, D; vs short and with claw-like tip); posterior part of conductor (Cc) bifurcated (Figs 2B, 3D; vs pocket-like); copulatory openings located in anterior half of epigyne (Figs 2D, 3F, vs near epigastral fold); spermathecae convoluted (Figs 2E, 3G, vs kidney-shaped).

#### Description.

Male holotype (Fig. 3A) total length 3.55. Carapace 1.69 long, 1.33 wide; opisthosoma 1.92 long, 1.10 wide. Eye sizes and interdistances: AME 0.06, ALE 0.14, PME 0.10, PLE, 0.11; AME–AME 0.05, AME–ALE 0.03, PME–PME 0.12, PME–PLE 0.08, ALE–PLE 0.03. MOA 0.24 long, anterior width 0.19, posterior width 0.31. Clypeus height 0.19. Chelicerae with three promarginal and four retromarginal teeth. Leg measurements: I 7.32 (2.06, 2.64, 1.62, 1.00); II 5.69 (1.68, 1.97, 1.27, 0.77); III 4.80 (1.38, 1.53, 1.18, 0.71); IV 6.14 (1.74, 2.06, 1.54, 0.80). Leg I longer than leg IV.

***Palp*** (Figs 2A–C, 3C, D). Patella as long as tibia. Tibia: ventral tibial apophysis (TA) originates from base of tibia, longer than tibia, distal half partially grooved and bent posteriorly; retrolateral apophysis (RTA) as long as cymbium, almost six times longer than maximal width. Posterior arm of conductor (CP) claw-like, anterior arm (CA) membranous and groove-like. Embolus long filiform, with a broad base, originating at 6 o’clock position, anterior part resting in long groove of conductor.

**Female** (paratype, Fig. 3B, SWUC-T-CI-08-02) total length 4.79. Carapace 1.74 long, 1.32 wide; opisthosoma 3.32 long, 2.41 wide. Eye sizes and interdistances: AME 0.08, ALE 0.13, PME 0.10, PLE, 0.12; AME–AME 0.05, AME–ALE 0.03, PME–PME 0.08, PME–PLE 0.03, ALE–PLE 0.04. MOA 0.29 long, anterior width 0.20, posterior width 0.34. Clypeus height 0.15. Chelicerae with three promarginal and four retromarginal teeth. Leg measurements: I 5.86 (1.75, 2.05, 1.26, 0.80); II 5.00 (1.47, 1.72, 1.09, 0.72); III 4.39 (1.27, 1.39, 1.06, 0.67); IV 5.74 (1.59, 1.97, 1.45, 0.73).

***Epigyne*** (Figs 2D–E, 3F–G). Epigynal plate 1.5 times wider than long. Copulatory openings located in anterior hood (Ah). Copulatory ducts long and complexly winding. Spermathecae convoluted.

#### Variation.

Females (*n = 8*): total length 3.60–5.19.

#### Distribution.

Known only from the type locality, Xizang, China (Fig. 6).

### ﻿ Altimella ngamring

Taxon classificationAnimaliaAraneaeCicurinidae

Wang & Zhang sp. nov.

A327281B-3FE5-550F-B7CA-7F681043F063

https://zoobank.org/E4E657ED-49D2-44C1-B5FB-293D4437F0DC

Figs 4 , 5 , 6 昂仁高朗蛛

#### Type material.

***Holotype*** ♂ (SWUC-T-CI-09-01), China, Xizang, Shigatse City, Ngamring County, Kaga Town, Nanma Village, 29°12'41′′N, 87°19'42′′E, elev. 4265 m, 21.07.2020, L.Y. Wang et al. leg. ***Paratypes***: 3♀ (SWUC-T-CI-09-02 to 04) with same data as for holotype (SWUC).

#### Etymology.

The specific name is derived from the type locality; a noun in apposition.

#### Diagnosis.

The new species differs from *A.nedong* Wang & Zhang, sp. nov. (Figs 2, 3) by the short ventral tibial apophysis, shorter than tibia width (Figs 4B, C, 5D, E; vs longer than tibia, tip with groove); the posterior part of conductor pocket-like (Figs 4B, 5D; vs claw-like); copulatory openings located posteriorly in a joint fovea and not hidden by hood (Figs 4D, 5F; vs located anteriorly and hidden by hood); spermathecae kidney-shaped (Figs 4F, 5G; vs convoluted).

#### Description.

Male holotype (Fig. 5A) total length 2.30. Carapace 1.09 long, 0.88 wide; opisthosoma 1.26 long, 0.88 wide. Eye sizes and interdistances: AME 0.05, ALE 0.08, PME 0.06, PLE, 0.08; AME–AME 0.03, AME–ALE 0.01, PME–PME 0.06, PME–PLE 0.05, ALE–PLE 0.02. MOA 0.16 long, anterior width 0.12, posterior width 0.18. Clypeus height 0.11. Chelicerae with three promarginal and three retromarginal teeth. Leg measurements: I 3.21 (0.90, 1.07, 0.70, 0.54); II 2.87 (0.84, 0.98, 0.60, 0.45); III 2.54 (0.73, 0.81, 0.56, 0.44); IV 3.24 (0.93, 1.01, 0.87, 0.43).

***Palp*** (Figs 4A–C, 5C, D). Tibia longer than patella. Ventral tibial apophysis (TA) shorter than the tibia, with hook-like tip; retrolateral apophysis (RTA) large and as long as cymbium with single fold; posterior arm of conductor (CP) pocket-like, lamelliform with sharp tip, anterior arm of conductor (CA) unobvious; embolus long, filiform, with a broad base, originating at 6 o’clock position, anterior part resting in the long groove of conductor.

**Female** (paratype, Fig. 5B, SWUC-T-CI-09-02) total length 2.96. Carapace 1.08 long, 0.85 wide; opisthosoma 1.87 long, 1.32 wide. Eye sizes and interdistances: AME 0.05, ALE 0.09, PME 0.06, PLE, 0.08; AME–AME 0.04, AME–ALE 0.02, PME–PME 0.05, PME–PLE 0.06, ALE–PLE 0.03. MOA 0.16 long, anterior width 0.15, posterior width 0.19. Clypeus height 0.08. Chelicerae with three promarginal and three retromarginal teeth. Leg measurements: I 2.88 (0.79, 1.05, 0.61, 0.43); II 2.59 (0.72, 0.87, 0.54, 0.46); III 2.33 (0.68, 0.65, 0.55, 0.45); IV 3.19 (0.93, 1.07, 0.75, 0.44).

***Epigyne*** (Figs 4D, E, 5F, G). Epigynal plate longer than wide. Copulatory openings located posteriorly in a wide fovea. Copulatory ducts long, C-shaped in dorsal view, together forming an X-shape. Spermathecae kidney-shaped. Fertilization ducts hook-like.

#### Variation.

Females (*n = 3*): total length 2.70–2.96.

#### Distribution.

Known only from the type locality, Xizang, China (Fig. 6).

## Supplementary Material

XML Treatment for Altimella

XML Treatment for Altimella nedong

XML Treatment for Altimella ngamring

## ﻿Additional information

### Author ORCIDs

Lu-Yu Wang https://orcid.org/0000-0002-5250-3473

Yan-Nan Mu https://orcid.org/0000-0002-2504-673X

Yong-Qiang Xu https://orcid.org/0000-0002-1889-9531

Feng Zhang https://orcid.org/0000-0002-3347-1031

Zhi-Sheng Zhang https://orcid.org/0000-0002-9304-1789

### Data availability

All of the data that support the findings of this study are available in the main text.
